# Simultaneous quantification of perfusion, permeability, and leakage effects in brain gliomas using dynamic spin-and-gradient-echo echoplanar imaging MRI

**DOI:** 10.1007/s00330-023-10215-z

**Published:** 2023-10-26

**Authors:** Francesco Sanvito, Catalina Raymond, Nicholas S. Cho, Jingwen Yao, Akifumi Hagiwara, Joey Orpilla, Linda M. Liau, Richard G. Everson, Phioanh L. Nghiemphu, Albert Lai, Robert Prins, Noriko Salamon, Timothy F. Cloughesy, Benjamin M. Ellingson

**Affiliations:** 1 UCLA Brain Tumor Imaging Laboratory (BTIL), Center for Computer Vision and Imaging Biomarkers, University of California Los Angeles, 924 Westwood Blvd, Los Angeles, CA 90024 USA; 2 Department of Radiological Sciences, David Geffen School of Medicine, University of California Los Angeles, 885 Tiverton Dr, Los Angeles, CA 90095 USA; 3 Unit of Radiology, Department of Clinical, Surgical, Diagnostic, and Pediatric Sciences, University of Pavia, Viale Camillo Golgi 19, 27100 Pavia, Italy; 4 Medical Scientist Training Program, David Geffen School of Medicine, University of California Los Angeles, 885 Tiverton Dr, Los Angeles, CA 90095 USA; 5 Department of Bioengineering, Henry Samueli School of Engineering and Applied Science, University of California Los Angeles, 7400 Boelter Hall, Los Angeles, CA 90095 USA; 6 Department of Radiology, Juntendo University School of Medicine, Bunkyo City, 2-Chōme-1-1 Hongō, Tokyo, 113-8421 Japan; 7 Department of Neurosurgery, David Geffen School of Medicine, University of California Los Angeles, 885 Tiverton Dr, Los Angeles, CA 90095 USA; 8 Department of Neurology, David Geffen School of Medicine, University of California Los Angeles, 885 Tiverton Dr, Los Angeles, CA 90095 USA; 9 Department of Psychiatry and Biobehavioral Sciences, David Geffen School of Medicine, University of California Los Angeles, 885 Tiverton Dr, Los Angeles, CA 90095 USA

**Keywords:** Glioblastoma, Magnetic resonance imaging, Perfusion imaging, Vascular permeability, Blood–brain barrier

## Abstract

**Objective:**

To determine the feasibility and biologic correlations of dynamic susceptibility contrast (DSC), dynamic contrast enhanced (DCE), and quantitative maps derived from contrast leakage effects obtained simultaneously in gliomas using dynamic spin-and-gradient-echo echoplanar imaging (dynamic SAGE-EPI) during a single contrast injection.

**Materials and methods:**

Thirty-eight patients with enhancing brain gliomas were prospectively imaged with dynamic SAGE-EPI, which was processed to compute traditional DSC metrics (normalized relative cerebral blood flow [nrCBV], percentage of signal recovery [PSR]), DCE metrics (volume transfer constant [*K*^trans^], extravascular compartment [*v*_*e*_]), and leakage effect metrics: Δ*R*_2,ss_* (reflecting T_2_*-leakage effects), Δ*R*_1,ss_ (reflecting T_1_-leakage effects), and the transverse relaxivity at tracer equilibrium (*TRATE*, reflecting the balance between Δ*R*_2,ss_* and Δ*R*_1,ss_). These metrics were compared between patient subgroups (treatment-naïve [TN] vs recurrent [R]) and biological features (IDH status, Ki67 expression).

**Results:**

In IDH wild-type gliomas (IDH^wt^—i.e., glioblastomas), previous exposure to treatment determined lower TRATE (*p *= 0.002), as well as higher PSR (*p *= 0.006), *K*^trans^ (*p *= 0.17), Δ*R*_1,ss_ (*p *= 0.035), *v*_*e*_ (*p *= 0.006), and ADC (*p *= 0.016). In IDH-mutant gliomas (IDH^m^), previous treatment determined higher *K*^trans^ and Δ*R*_1,ss_ (*p *= 0.026). In TN-gliomas, dynamic SAGE-EPI metrics tended to be influenced by IDH status (*p* ranging 0.09–0.14). *TRATE* values above 142 mM^−1^s^−1^ were exclusively seen in TN-IDH^wt^, and, in TN-gliomas, this cutoff had 89% sensitivity and 80% specificity as a predictor of Ki67 > 10%.

**Conclusions:**

Dynamic SAGE-EPI enables *simultaneous* quantification of brain tumor perfusion and permeability, as well as mapping of novel metrics related to cytoarchitecture (*TRATE*) and blood–brain barrier disruption (Δ*R*_1,ss_), with a single contrast injection.

**Clinical relevance statement:**

Simultaneous DSC and DCE analysis with dynamic SAGE-EPI reduces scanning time and contrast dose, respectively alleviating concerns about imaging protocol length and gadolinium adverse effects and accumulation, while providing novel leakage effect metrics reflecting blood–brain barrier disruption and tumor tissue cytoarchitecture.

**Key Points:**

*• Traditionally, perfusion and permeability imaging for brain tumors requires two separate contrast injections and acquisitions.*

*• Dynamic spin-and-gradient-echo echoplanar imaging enables simultaneous perfusion and permeability imaging.*

*• Dynamic spin-and-gradient-echo echoplanar imaging provides new image contrasts reflecting blood–brain barrier disruption and cytoarchitecture characteristics.*

**Graphical Abstract:**

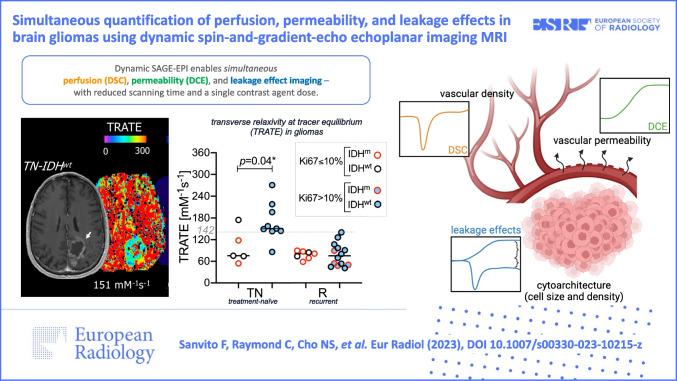

**Supplementary Information:**

The online version contains supplementary material available at 10.1007/s00330-023-10215-z.

## Introduction

Brain gliomas are characterized by heterogeneous prognosis, depending on biological and molecular features [1, 2] and on a variable response to treatment [3–5]. Aggressive tumors rely on a more intense neoangiogenesis, resulting in a dysfunctional neovasculature with blood–brain barrier (BBB) breakdown [6–9]. Magnetic resonance imaging (MRI) can non-invasively quantify vascularization and BBB permeability in vivo, using two separate techniques based on contrast agent (CA) administration: dynamic susceptibility contrast (DSC), a T_2_*-weighted gradient-echo (GE) sequence, and dynamic contrast enhanced (DCE), a T_1_-weighted sequence [10]. While DSC perfusion imaging yields rCBV (relative cerebral blood volume), reflecting vascular density [11], DCE permeability imaging allows to compute *K*^trans^ (volume transfer constant), representing the rate of CA leakage and therefore BBB permeability [12]. These techniques can aid glioma grading [13–15], molecular profiling [16–19], differential diagnosis [10, 20–24], and the distinction between treatment effects and tumor recurrence [10, 24–27], and have become part of the clinical brain tumor work-up in many neuroimaging centers [28]. However, performing perfusion (DSC) and permeability (DCE) imaging requires two separate acquisitions, which increases scanning time, and two full CA doses, which raises concerns for chronic gadolinium deposition [29] and adverse effects in patients with impaired renal function [30].

Dual-echo DSC simultaneously acquires two GE echoes, which can be processed to disentangle the T_2_*- and T_1_-contributions that coexist in a DSC sequence [31]. In fact, DSC bears some T_1_-weighting, which can be sorted out by analyzing two GE echoes. The T_1_-contribution can further be used for a DCE analysis, enabling complementary permeability imaging without extra scanning time and without a second CA dose [31].

Additionally, dual-echo DSC allows to compute quantitative maps derived from CA leakage effects. In the presence of BBB breakdown, CA leaks from the intravascular (IV) to the extravascular extracellular (EEC) compartment, resulting in competing T_2_*- and T_1_-leakage effects, whose balance is influenced by tissue-related factors [32, 33]. In a traditional single-echo DSC, the overall balance between T_2_*- and T_1_-leakage effects can be evaluated with a metric named percentage of signal recovery (PSR) [34], which is valuable for differential diagnosis because tissue-related factors differ among tumor types (gliomas, lymphomas, and meningiomas) [34, 35]. However, PSR strongly depends on acquisition parameters such as flip angle (FA) and echo time (TE) [36], a major obstacle when generalizing reliable PSR cutoffs across institutions. Moreover, PSR only provides overall estimates of the balance between T_2_*- and T_1_-leakage effects, which cannot be disentangled with a single-echo DSC. Conversely, dual-echo DSC allows to *separately* evaluate T_1_ and T_2_* contributions, and to compute a novel quantitative biomarker named transverse relaxivity at tracer equilibrium (*TRATE*), which quantifies T_2_*-leakage effects normalized to the estimated CA concentration (derived from T_1_-leakage effects) [32, 33]. Despite bearing similar information to PSR, *TRATE* is independent from acquisition factors (FA and TE) [33]. Results from simulated and preclinical data advocate for *TRATE* as a biomarker for cytoarchitectural features such as cell volume fraction and cell size, but its application on human brain tumors has only been preliminary explored in five recurrent high-grade gliomas [33].

In this study, we aim to *simultaneously* obtain perfusion, permeability, and novel leakage effect maps in a cohort of human gliomas, both newly diagnosed and recurrent, using a dynamic spin-and-gradient-echo echoplanar imaging (dynamic SAGE-EPI) acquisition. In fact, the first and second echoes of a dynamic SAGE-EPI can serve as a dual-echo DSC sequence. First, we hypothesize that *TRATE* will correlate with PSR, and that Δ*R*_1,ss_ (quantifying T_1_-leakage effects) will correlate with *K*^trans^, since these metrics are considered quantitative markers for cytoarchitecture (*TRATE* and PSR) and BBB permeability (Δ*R*_1,ss_ and *K*^trans^), respectively. Second, we hypothesize that increased *TRATE* will reflect aggressive cytoarchitectural features, and will therefore be higher in tumors with higher expression of Ki67 (a marker of cell proliferation) and with IDH wild-type (IDH^wt^) status (i.e., glioblastomas).

## Materials and methods

### Patient selection

Patients who gave informed written consent to join the research studies approved by our institutional review board (IRB#14-001261 and #21-000514) were imaged prospectively at our institution. At the time of the study, IRB#14-001261 included patients acquired from April 2015 to October 2020, while IRB#21-000514 from October 2021 to June 2022. Inclusion criteria for the present study were as follows: enhancing lesion, availability of dynamic SAGE-EPI datasets, surgical resection *after* dynamic SAGE-EPI, availability of surgical pathological reports, histopathological diagnosis of adult-type diffuse glioma (i.e., astrocytoma, oligodendroglioma, or glioblastoma) [1].

### Magnetic resonance imaging

Magnetic resonance imaging data was collected using a 3-T Siemens Prisma (Siemens Healthineers) according to the standardized brain tumor imaging protocol [37], including pre- and post-contrast T_1_-weighted images at 1-mm isotropic resolution, T_2_-weighted FLAIR images with 3-mm slice thickness, diffusion tensor imaging (DTI) with 2-mm isotropic resolution obtained in 64 directions with *b*-values = 1000 s/mm^2^, and a single *b *= 0 image. The apparent diffusion coefficient (ADC) was estimated from the mean diffusivity computed from the DTI tensor on the scanner. A custom dynamic SAGE-EPI sequence (patent: US 11,378,638 B2) [11, 38, 39] was acquired during injection of a single dose of Gadavist^®^ (Gadobutrol, Bayer) (~ 0.1 mL/kg) at a rate of ~ 4 mL/s, according to guidelines [40]. Dynamic SAGE-EPI was acquired using two gradient echoes (echo 1 with TE_1 _= 14 ms, echo 2 with TE_2 _= 34.1 ms), an asymmetric spin echo (TE_3 _= 58.0 ms), and a spin echo (TE_4 _= 92.4 ms), with a repetition time (TR) = 2000 ms, matrix size = 240 × 218 mm, GRAPPA = 3, voxel size 1.875 × 1.875 × 5 mm, 19 axial slices, and 90 repetitions.

### Image analysis

**Fig. 1:**
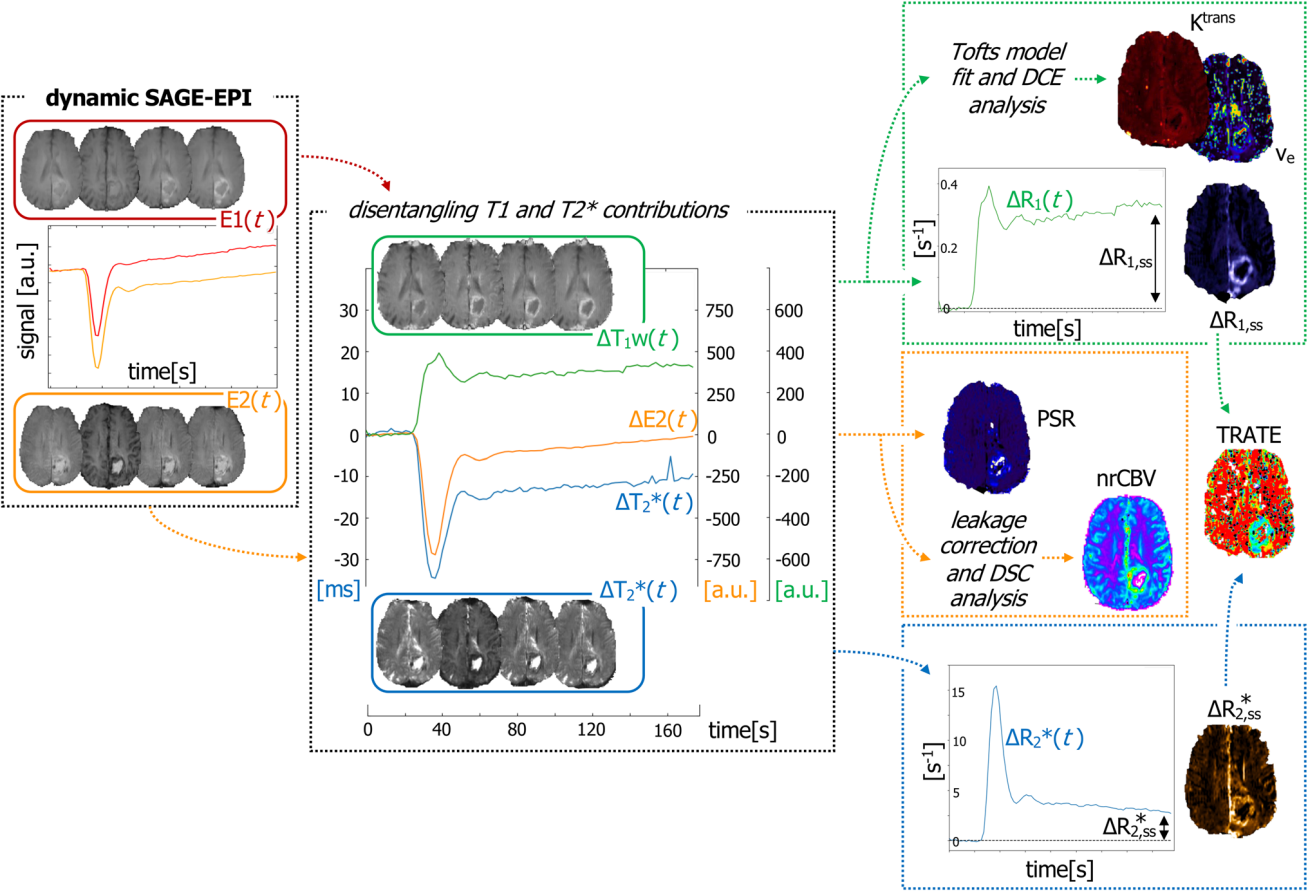
Image processing pipeline of dynamic SAGE-EPI to obtain MRI quantitative maps. From echo 1 (E1, red, a GE with TE = 14 ms) and echo 2 (E2, orange, a GE with TE = 34.1 ms) of the dynamic SAGE-EPI datasets, the T_1_ (green) and T_2_* (blue) contributions to DSC signals were disentangled. The T_1_ contribution was used for DCE analysis and to quantify Δ*R*_1,ss_, while the T_2_* contribution was used to quantify Δ*R*_2,ss_*. *TRATE* was generated from Δ*R*_2,ss_* and Δ*R*_1,ss_. The classic DSC metrics were computed from E2

Dynamic SAGE-EPI was processed according to the pipeline in Fig. 1. The first GE (echo 1: E1) and second GE (echo 2: E2) were separated, and motion-corrected using FSL (University of Oxford, https://fsl.fmrib.ox.ac.uk/fsl/) *mcflirt* function. The changes in transverse relaxation rate over time compared to baseline (Δ*R*_2_*(*t*) curve, [s^−1^]), quantifying T_2_*-contribution (Suppl. Eq. 1), and T_1_-contribution over time (T_1_w(*t*)) [arbitrary units] (Suppl. Eq. 2) were obtained voxel-wise as illustrated in Stokes et al [31], where T_1_w(*t*) is the extrapolated signal for TE = 0 ms. For visualization, ΔT_2_*(*t*) [s] and ΔT_1_w(*t*) [arbitrary units] curves were also obtained (Suppl. Eq. 3). To quantify T_1_ effects, the change in longitudinal relaxation rate over time compared to baseline (ΔR_1_(*t*) curve, [s^−1^]) was computed on a voxel-wise basis from T_1_w(*t*) according to the equations from the Quantitative Imaging Biomarkers Alliance (QIBA, https://www.rsna.org/research/quantitative-imaging-biomarkers-alliance), assuming a fixed T_1_ (T_10_) of 1.4s for tissue, as proposed in Conte et al [41] (Suppl. Eq. 4). An estimated CA concentration over time (C(*t*)) [mM^−1^] was obtained by normalizing Δ*R*_1,ss_ to the longitudinal relaxivity of Gadobutrol at 3T (r_1_), set to 5.0 mM^−1^s^−1^ as computed by Rohrer et al [42] (Suppl. Eq. 5) and reported by the American College of Radiology (https://www.acr.org).

Δ*R*_2,ss_* (Δ*R*_2_* at steady state), Δ*R*_1,ss_ (Δ*R*_1_ at steady state), and *C*_ss_ (CA concentration at steady state) voxel-wise maps were computed by averaging the final 10 timepoints of the Δ*R*_2_*(*t*), Δ*R*_1_(*t*), and *C*(*t*) time curves, respectively [33]. Δ*R*_2,ss_* and *C*_ss_ were combined to compute the transverse relaxivity at tracer equilibrium (*TRATE* [mM^−1^s^−1^]), corresponding to T_2_*-leakage effects normalized to CA concentration (Suppl. Eq. 5) [33]. *TRATE*, Δ*R*_2,ss_*, and Δ*R*_1,ss_ will be referred to as “leakage effect maps.”

Normalized rCBV maps (nrCBV) were generated from E2 with a bidirectional leakage correction algorithm [43] with subsequent normalization to the cerebral median rCBV. PSR maps were generated from E2 without leakage correction, as described in Lee et al [35].

For DCE analysis, a Tofts model [12] was fit to T_1_w(*t*) (assuming T_10_ and r_1_ as above) to compute voxel-wise *K*^trans^ and *v*_*e*_ (extracellular volume) maps, by adapting the open-access OSIPI DCE code (https://osipi.org/). A region of interest (ROI) was placed in the superior sagittal sinus to extract the arterial input function (AIF). Unlike DSC, the proposed DCE analysis is *not* “traditional,” as it is based on T_1_w(*t*) computed from dynamic SAGE-EPI, as opposed to acquired T_1_-weighted datasets.

All maps were registered to post-contrast T1 with the FSL *flirt* function.

### Segmentation and quality check

Pre- and post-contrast T_1_-weighted images were co-registered, normalized, and voxel-by-voxel subtracted to obtain T_1_-weighted subtraction maps, as described in Ellingson et al [5]. Voxels with a ≥ 10% increase in normalized T_1_ signal after CA administration were isolated within the lesion area and included in the enhancing tumor segmentation. A neuroradiologist with 7 years of experience in neuroimaging (F.S.) quality-checked maps, registrations, segmentations, AIF-ROIs, and Tofts fits.

### Clinical and pathological information

The patients’ clinical records and pathology reports were reviewed in order to retrieve the following information: sex category, age, previous exposure to treatment, tumor grade and molecular status, Ki67 expression.

### Statistical analyses

Median values of MRI metrics were extracted from the tumor segmentation. The linear correlation between continuous variables was assessed with a correlation coefficient, interpreted as in previous literature [44, 45]. Group differences were assessed with Mann-Whitney *U* tests. Ki67 expression was binarized as ≤ 10% or > 10% as validated in previous studies [46, 47]. The significant *p*-value threshold was set to *p *< 0.05.

## Results

### Patients’ cohort characteristics

**Fig. 2:**
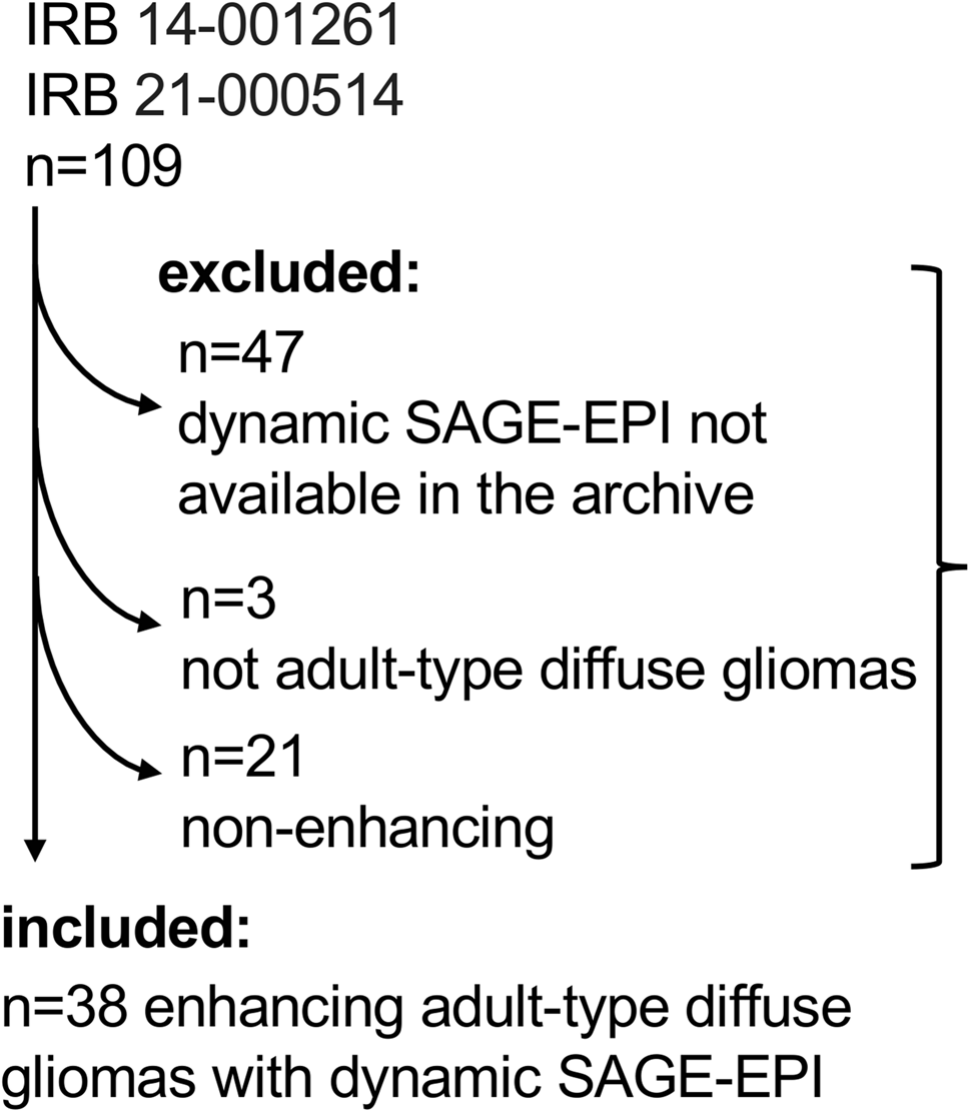
Flowchart of patients included and excluded from the study

Thirty-eight patients met the inclusion criteria (Fig. 2): fourteen treatment-naïve (TN) and twenty-four recurrent (R). Demographic, clinical, and pathological features of the cohort are summarized in Table 1. Suppl. Fig. 1 presents an overview of conventional MRI appearances of representative cases with various grades and treatment statuses.

**Table 1:** Demographic, clinical, and pathological features of the patients’ cohort

	Treatment-naïve *n *= 14	Recurrent *n *= 24
Age (years)	61.8 ± 12.9	50.1 ± 13.1
Sex category (F)	4 (28.6%)	7 (29.2%)
Main location		
Frontal	3 (21.4%)	8 (33.3%)
Parietal	3 (21.4%)	5 (20.8%)
Temporal	5 (35.7%)	4 (16.7%)
Multiple lobes	1 (7.1%)	5 (20.8%)
WM/deep GM	2 (14.3%)	2 (8.3%)
Grade		
2	1 (7.1%)	3 (12.5%)
3	1 (7.1%)	3 (12.5%)
4	12 (85.7%)	18 (75%)
Ki67 expression		
≤ 10%	5 (35.7%)	7 (29.2%)
> 10%	9 (64.3%)	14 (58.3%)
Not tested	0 (0%)	3 (12.5%)
IDH and 1p/19q		
IDH^m^ 1p/19q^int^	1 (7.1%)	9 (37.5%)
IDH^m^ 1p/19q^cod^	1 (7.1%)	2 (8.3%)
IDH^wt^	12 (85.7%)	13 (54.2%)
MGMT promoter		
Methylated (> 2%)	7 (50%)	12 (50%)
Unmethylated	7 (50%)	11 (45.8%)
Not tested	0 (0%)	1 (4.2%)
EGFR		
Amplified	5 (37.5%)	8 (33.3%)
Non-amplified	8 (57.1%)	11 (45.8%)
Not tested	1 (7.1%)	5 (20.8%)

Age is expressed as mean ± standard deviation. *WM*, white matter; *GM*, gray matter; *IDH*^*m*^, IDH-mutant; *IDH*^*wt*^, IDH wild-type; *1p/19q*^*cod*^, 1p/19q codeleted; *1p/19q*^*int*^, 1p/19q intact

### Relationships among MRI metrics

**Fig. 3:**
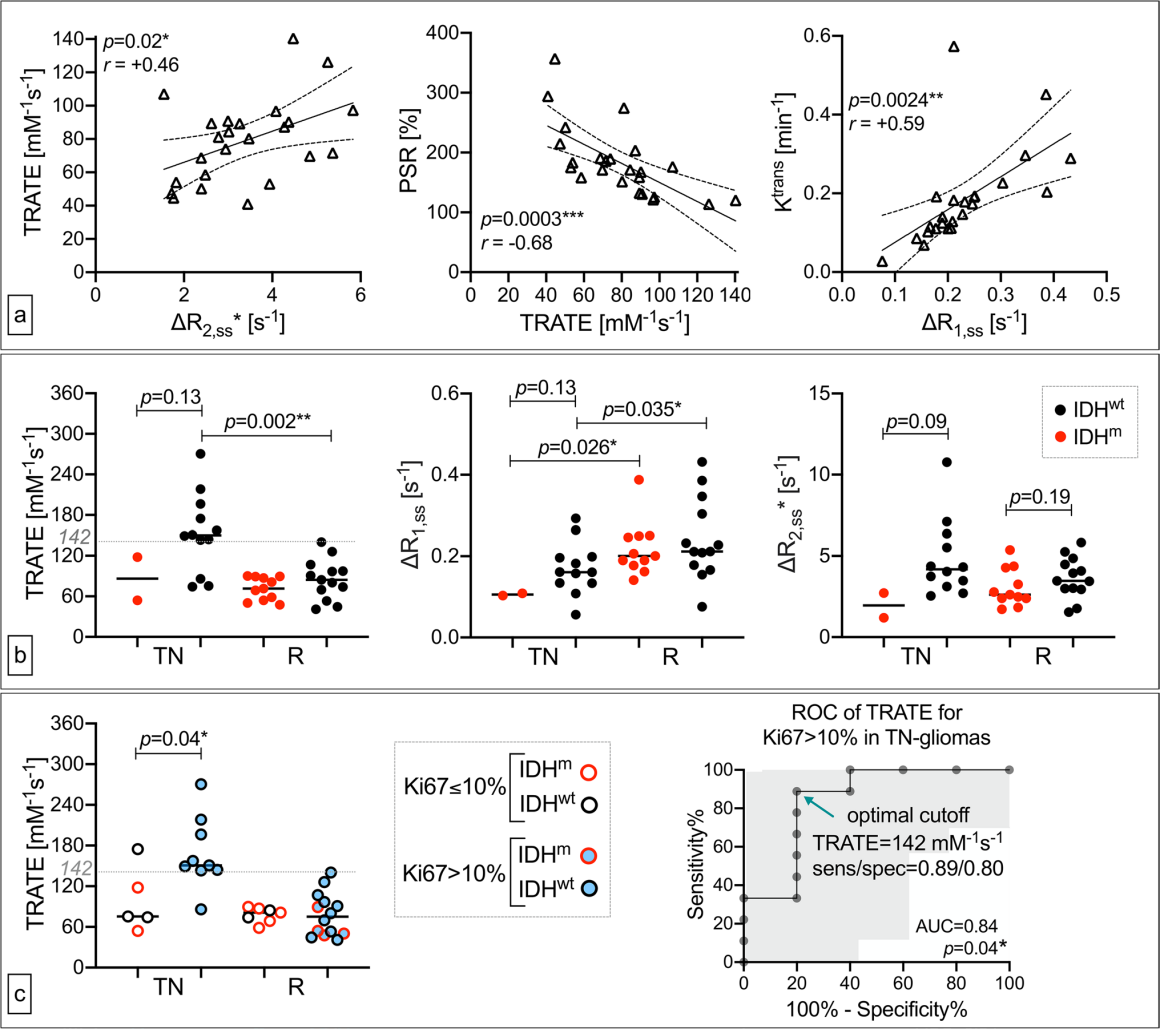
Correlations among MRI metrics and group differences. Panel **a** shows selected correlations among MRI metrics in R-gliomas, the most numerous subcohort. *TRATE* values depend on Δ*R*_2,ss_* and are inversely correlated with PSR values; Δ*R*_1,ss_ values are positively correlated with *K*^trans^. Panel **b** illustrates group differences in leakage effect metrics according to prior exposure to treatment and IDH status (red vs black). *TRATE* values were higher in TN-IDH^wt^ gliomas compared to their recurrent counterparts, due to comparable Δ*R*_2,ss_* values in the presence of lower Δ*R*_1,ss_ values. TN-IDH^wt^ gliomas tended to display higher *TRATE* values also compared to IDH^m^ gliomas, ascribable to higher Δ*R*_2,ss_* values. Notably, exclusively TN-IDH^wt^ showed median *TRATE* values > 142 mM^−1^s^−1^, and only *n *= 3 TN-IDH^wt^ lesions displayed low *TRATE* (< 142 mM^−1^s^−1^). Panel **c** displays *TRATE* differences according to Ki67 expression (white vs blue central color, while the border color reflects IDH status). In TN-gliomas, high Ki67 expression corresponded to higher *TRATE* values. *TRATE* had good diagnostic accuracy (AUC = 0.84) in distinguishing low and high Ki67 gliomas, and a cutoff of > 142 mM^−1^s^−1^ yielded sensitivity/specificity of 0.89/0.80 for prediction of Ki67 > 10% (see ROC curve with 95% CI). Notably, out of the *n *= 3 TN-IDH^wt^ lesions with low *TRATE* (**b**), *n *= 2 had low Ki67 (**c**). TN, treatment-naïve; R, recurrent; IDH^m^, IDH-mutant; IDH^wt^, IDH wild-type. *p < 0.05, **p < 0.01, ***p < 0.001, ****p < 0.0001

Correlations among MRI metrics are displayed in Table 2 and Fig. 3a.

**Table 2:** Correlations between TRATE and perfusion, permeability, leakage effect, and diffusion MRI metrics

MRI metrics		Treatment-naïve			Recurrent		
		*p*	*r*	*R*^2^	*p*	*r*	*R*^2^
TRATE	Δ*R*_1,ss_	0.32			0.04*	− 0.42	0.18
TRATE	Δ*R*_2,ss_*	0.004**	+ 0.71	0.51	0.02*	+ 0.46	0.21
TRATE	PSR	< 0.0001****	− 0.87	0.76	0.0003***	− 0.68	0.46
TRATE	nrCBV	0.02*	+ 0.60	0.36	0.76		
TRATE	*K*^trans^	0.77			0.81		
TRATE	*v*_*e*_	0.31			0.86		
TRATE	ADC	0.04*	− 0.54	0.29	0.12	− 0.33	0.11

*p*, *p*-value; *r*, Pearson’s coefficient; *R*^2^, coefficient of determination. *r* and *R*^2^ are displayed only for correlations that are either significant or trend towards significance. *p < 0.05, **p < 0.01, ***p < 0.001, ****p < 0.0001

*TRATE* values strongly correlated with Δ*R*_2,ss_* (*p *= 0.004, *r *= + 0.71) but and not with Δ*R*_1,ss_ (*p *= 0.32) in TN-gliomas, while moderately correlated with both Δ*R*_2,ss_* (*p *= 0.02, *r *= + 0.46; Fig. 3a) and Δ*R*_1,ss_ (*p *= 0.04, *r *= − 0.42) in R-gliomas. *TRATE* values correlated with ADC (*p *= 0.04, *r *= − 0.54) and nrCBV (*p *= 0.02, *r *= + 0.60) in TN-gliomas, but not in R-gliomas. As expected, *TRATE* strongly correlated with PSR (TN/R: *p *< 0.0001/*p *= 0.0003, *r *= − 0.87/*r *= − 0.68; Fig. 3a).

Similarly to *TRATE*, PSR values depended on Δ*R*_2,ss_* in both TN- (*p *= 0.02, *r *= − 0.61) and R-gliomas (*p *= 0.03, *r *= − 0.45), but not on Δ*R*_1,ss_. Like *TRATE*, in TN-gliomas, PSR correlated with ADC (*p *= 0.015, *r *= + 0.63) and nrCBV (*p *= 0.005, *r *= − 0.70).

Δ*R*_1,ss_ strongly correlated with *K*^trans^ in both TN-gliomas (*p *= 0.036, *r *= + 0.68) and R-gliomas (*p *= 0.0024, *r *= + 0.59; Fig. 3a).

### Group differences based on treatment status, IDH status, and Ki67 expression

Differences based on treatment status are shown in Table 3 and Fig. 3b. TN-gliomas had lower Δ*R*_1,ss_ (IDH^m^/IDH^wt^: *p *= 0.026/0.035) and *K*^trans^ (IDH^m^/IDH^wt^: *p *= 0.026/0.17) than R-gliomas overall, which reflect a lower EEC concentration of CA and a slower CA leakage rate, respectively. In TN-IDH^wt^, Δ*R*_2,ss_* was comparable to R-IDH^wt^ despite the EEC CA being less concentrated, which resulted in significantly higher *TRATE* values (*p *= 0.002; Fig. 3b). *TRATE* > 142 mM^−1^s^−1^ was exclusively seen in TN-IDH^wt^ (Fig. 3b). TN-IDH^wt^ also had significantly lower PSR (*p *= 0.006), *v*_*e*_ (*p *= 0.006), and ADC (*p *= 0.016) than R-IDH^wt^.

**Table 3:** Differences in MRI metrics based on previous exposure to treatment and IDH status

MRI metrics	Treatment-naïve		Recurrent		*p*-values			
	*TN-IDH*^*m*^	*TN-IDH*^*wt*^	*R-IDH*^*m*^	*R-IDH*^*wt*^	*TN: IDH*^*m*^* vs IDH*^*wt*^	*R: IDH*^*m*^* vs IDH*^*wt*^	*IDH*^*m*^*: TN vs R*	*IDH*^*wt*^*: TN vs R*
TRATE [mM^−1^s^−1^]	86.1 ± 45.1	153.4 ± 58.3	71.6 ± 16.9	85.1 ± 29.6	0.13	0.25	0.64	0.002**
Δ*R*_1,ss_ [s^−1^]	0.11 ± 0.004	0.17 ± 0.06	0.22 ± 0.07	0.24 ± 0.10	0.13	0.57	0.026*	0.035*
Δ*R*_2,ss_* [s^−1^]	2.0 ± 1.0	4.8 ± 2.3	3.0 ± 1.2	3.7 ± 1.3	0.09	0.19	0.41	0.25
PSR [%]	172 ± 65	121 ± 30	192 ± 41	177 ± 72	0.13	0.14	0.78	0.006**
nrCBV	0.72 ± 0.58	2.15 ± 0.74	1.20 ± 0.36	2.17 ± 0.97	0.09	0.02*	0.15	0.93
*K*^trans^ [min^−1^]	0.06 ± 0.006	0.16 ± 0.12	0.14 ± 0.04	0.22 ± 0.15	0.09	0.11	0.026*	0.17
*v*_*e*_	0.48 ± 0.07	0.28 ± 0.14	0.34 ± 0.14	0.49 ± 0.18	0.14	0.055	0.23	0.006**
ADC [× 10^−6^ mm^2^/s]	1118 ± 107	977 ± 240	1227 ± 155	1228 ± 199	0.35	0.95	0.51	0.016*

Group values for MRI metrics are reported as mean ± standard deviation. *p < 0.05, **p < 0.01, ***p < 0.001, ****p < 0.0001. *TN*, treatment-naïve; *R*, recurrent; *IDH*^*m*^, IDH-mutant; *IDH*^*wt*^, IDH wild-type

Differences based on IDH status are shown in Table 3 and Fig. 3b. TN-IDH^wt^ tended to have more pronounced CA leakage (higher Δ*R*_1,ss_ and *K*^trans^) than TN-IDH^m^ (*p *= 0.13/0.09), as well as higher Δ*R*_2,ss_* (*p *= 0.09). Since Δ*R*_2,ss_* differences greatly exceeded Δ*R*_1,ss_ differences, *TRATE* showed a trend towards being higher in TN-IDH^wt^ than in TN-IDH^m^ (*p *= 0.13), and TN-IDH^m^ displayed *TRATE* values comparable to R-gliomas (Table 3; Fig. 3b). In TN, also PSR (*p *= 0.13), *v*_*e*_ (*p *= 0.14), and nrCBV (*p *= 0.09) tended to differ depending on IDH status. The low sample size in the TN-IDH^m^ subgroup is probably a reason for such trends not being statistically significant. Notably, nrCBV was the only metric with significantly different values based on IDH status in the recurrent setting.

Differences based on Ki67 expression are shown in Table 4 and Fig. 3c. TN-gliomas with high Ki67 expression had higher *TRATE* (*p *= 0.04), higher nrCBV (*p *= 0.001), and lower PSR values (*p *= 0.04) than low Ki67 lesions (Table 4; Fig. 3c). In TN-gliomas, *TRATE* predicted a high Ki67 (> 10%) expression with AUC = 0.84, and a cutoff of *TRATE *> 142 mM^−1^s^−1^ corresponded to sensitivity and specificity of 89% and 80% (Fig. 3c). Notably, out of *n *= 3 TN-IDH^wt^ with low *TRATE* (< 142 mM^−1^s^−1^), *n *= 2 had low Ki67 (Fig. 3b, c).

**Table 4:** Differences in MRI metrics based on Ki67 expression

MRI metrics	Treatment-naïve			Recurrent		
	*Ki67 ≤ 10%*	*Ki67 > 10%*	*p*	*Ki67 ≤ 10%*	*Ki67 > 10%*	*p*
TRATE [mM^−1^s^−1^]	99.3 ± 48.2	168.5 ± 53.0	0.04*	77.6 ± 11.2	77.9 ± 31.8	0.86
Δ*R*_1,ss_ [s^−1^]	0.19 ± 0.09	0.14 ± 0.04	0.36	0.19 ± 0.04	0.23 ± 0.10	0.44
Δ*R*_2,ss_* [s^−1^]	3.8 ± 2.2	4.8 ± 2.6	0.60	2.9 ± 0.6	3.2 ± 1.2	0.58
PSR [%]	159 ± 47	111 ± 16	0.04*	189 ± 45	186 ± 70	0.53
nrCBV	1.02 ± 0.45	2.46 ± 0.55	0.001**	1.23 ± 0.73	1.81 ± 0.97	0.11
*K*^trans^ [min^−1^]	0.20 ± 0.17	0.12 ± 0.05	0.70	0.13 ± 0.04	0.21 ± 0.15	0.25
*v*_*e*_	0.37 ± 0.12	0.28 ± 0.16	0.24	0.37 ± 0.15	0.45 ± 0.19	0.36
ADC [× 10^−6^ mm^2^/s]	1107 ± 256	936 ± 200	0.19	1293 ± 183	1202 ± 176	0.44

Group values for MRI metrics are reported as mean ± standard deviation, *p* = *p*-value. *p < 0.05, **p < 0.01, ***p < 0.001, ****p < 0.0001. *TN*, treatment-naïve; *R*, recurrent; *IDH*^*m*^, IDH-mutant; *IDH*^*wt*^, IDH wild-type

### Representative cases

**Fig. 4:**
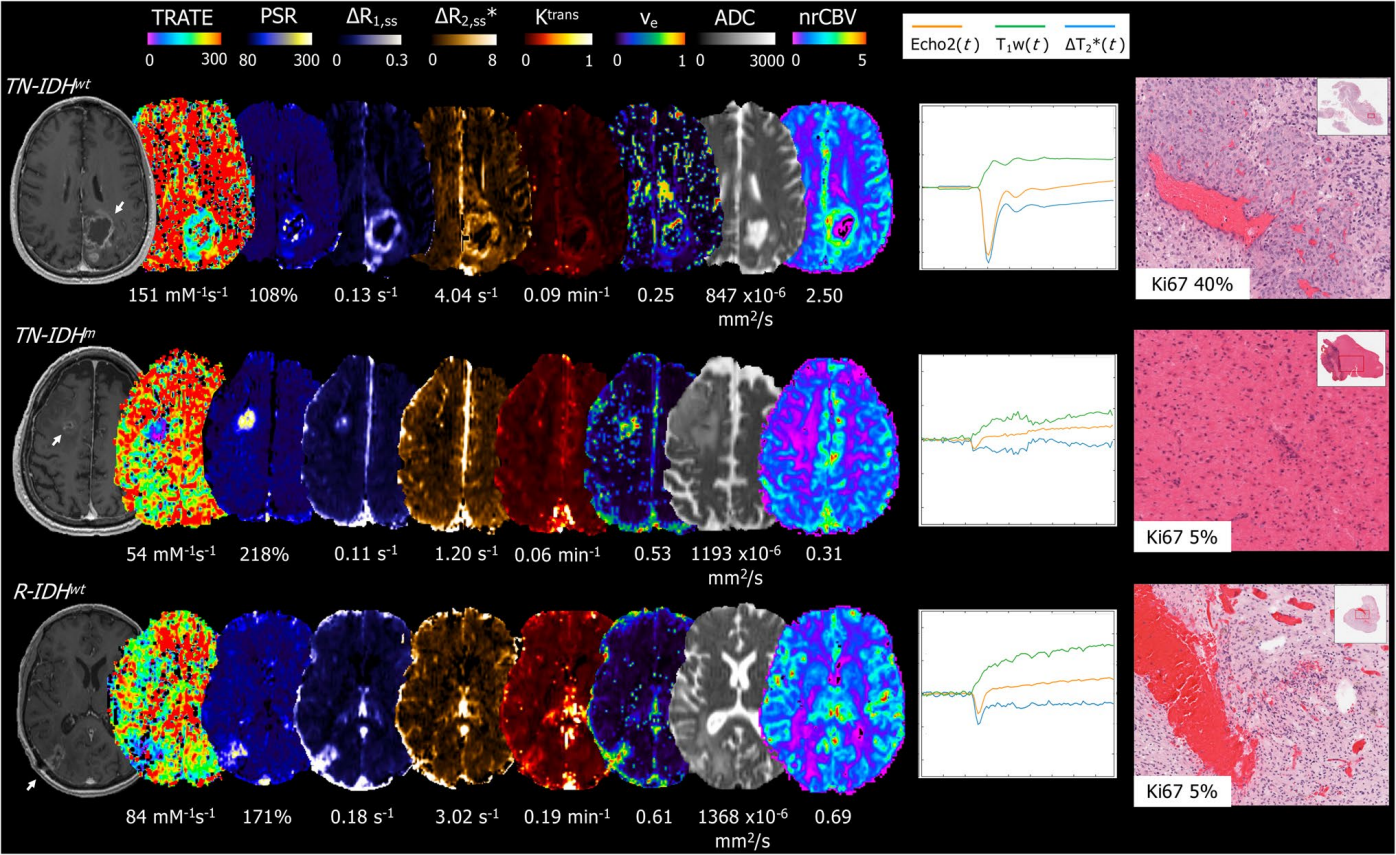
Representative cases. Perfusion, permeability, and leakage effect maps derived from dynamic SAGE-EPI in three enhancing gliomas (TN-IDH^wt^, TN-IDH^m^, and R-IDH^wt^, respectively) are displayed along with the disentangled T_1_ (green line) and T_2_* (blue line) signal contributions, with histopathological H&E slides, and with their appearance on post-contrast T_1_-weighted images (arrows). The TN-IDH^wt^ glioma (top row) displays higher *TRATE* values, driven by more pronounced T_2_*-leakage effects (Δ*R*_2,ss_* map and blue line) compared to T1-leakage effects (Δ*R*_1,ss_ map and green line). Histopathology showed high cellularity, microvascular proliferation, and high Ki67 expression. The TN-IDH^m^ glioma (middle row) displays lower *TRATE* values, as well as lower cell density and Ki67. The R-IDH^wt^ glioma (bottom row) shows lower *TRATE* due to similar T_2_*-leakage effects (Δ*R*_2,ss_* map and blue line) in the presence of more prominent T_1_-leakage effects (Δ*R*_1,ss_ map and green line). Histopathology showed recurrent tumor mixed with treatment effects, including reactive gliosis, hyalinized vessels, and foci of necrosis. TN, treatment-naïve; R, recurrent; IDH^m^, IDH-mutant; IDH^wt^, IDH wild-type

Figure 4 displays perfusion, permeability, and leakage effect MRI maps computed from dynamic SAGE-EPI for representative patients, as well as the disentangled T_1_ and T_2_* signal contributions, and histopathological images.

## Discussion

This study demonstrates the feasibility of computing perfusion (DSC), permeability (DCE), and quantitative maps derived from contrast leakage effects from a *single* dynamic SAGE-EPI sequence with a *single bolus* of contrast agent. Additionally, this study demonstrated that such leakage effect metrics (i.e., Δ*R*_1,ss_, Δ*R*_2,ss_*, *TRATE*) in gliomas depend on previous exposure to treatment, IDH status, and Ki67 expression. While simultaneous DSC and DCE were already proposed in human patients by Stokes et al [31] and *TRATE* computation was originally proposed mainly in the preclinical setting by Semmineh et al [33], this is the first study proposing a pipeline to *simultaneously* compute DSC, DCE, and leakage effect maps in human gliomas, and assessing their biological correlates.

This approach has multiple clinical benefits. First, dynamic SAGE-EPI is 3 minutes long (extending to 5–6 min may be considered—see the limitations section), while separate DSC and DCE would require at least 10 min of total scanning time. This is clinically relevant because brain tumor patients already undergo very time-consuming protocols, including multiple advanced and functional sequences [17, 48], which are a burden for patients. Second, simultaneous acquisition eliminates the need of a second bolus of contrast agent (CA). Double-dose CA raises concerns for chronic gadolinium deposition in deep gray matter [29] and for adverse effects in patients with impaired renal function [30], especially considering that brain tumor patients undergo serial follow-up MRI with CA. Dynamic SAGE-EPI would allow to perform DSC and DCE at every timepoint with a remarkable cumulative reduction of administered CA. Third, our pipeline allows the quantification of leakage effects, which provide further complementary insights into vascular permeability and tissue cytoarchitecture, as further discussed.

In the present study, Δ*R*_1,ss_, which is thought to be proportional to CA concentration in the extravascular extracellular compartment (EEC) [32, 33], was found to correlate with *K*^trans^, representing the rate of CA leakage from the intravascular (IV) compartment to EEC [10, 12]. The exposure to previous treatment was associated both with higher Δ*R*_1,ss_ and *K*^trans^, reflecting a more prominent and faster CA leakage in EEC. This is consistent with the well-established notion that radiation increases blood–brain barrier (BBB) permeability [49–51]. These two findings advocate for Δ*R*_1,ss_ as a quantitative biomarker of BBB breakdown, and suggest that it could be a surrogate of *K*^trans^. This is relevant because *K*^trans^ values are dramatically affected by the AIF selection and the pharmacokinetic model fit [52], which leads to highly variable measurements. For instance, average *K*^trans^ values [min^−1^] in glioblastoma cohorts ranged 0.035–1.8 across studies [21, 31, 53–55] (0.16 in this study). Conversely, Δ*R*_1,ss_ [s^−1^] is a simpler metric, independent from model fit or AIF. Therefore, Δ*R*_1,ss_, if further validated, would constitute a more universal quantitative biomarker for blood–brain barrier (BBB) breakdown.

*TRATE* showed characteristic high values in treatment-naïve (TN) IDH^wt^, which were the only tumors displaying *TRATE *> 142 mM^−1^s^−1^. Additionally, *TRATE *> 142 mM^−1^s^−1^ in TN-gliomas predicted high Ki67 expression with good diagnostic performance (sensitivity/specificity: 0.89/0.80), and the few TN-IDH^wt^ with low *TRATE* values almost entirely had low Ki67 expression. Preclinical and simulated data by Semmineh et al [33] suggest that *TRATE* may be a cytoarchitectural biomarker, displaying higher values in the presence of higher cell volume fraction and/or larger cell size. Our results, taken together, are consistent with this interpretation. Higher *TRATE* values in IDH^wt^ gliomas (i.e., glioblastomas, as per 2021 WHO classification [1]) are consistent with their established higher cell density and proliferation rate, compared to lower grades [56]. As for Ki67 expression, while it does not directly represent cell density nor cell size, it is a biomarker of active cell proliferation [46, 47], and it is reasonable to speculate that gliomas with higher proliferation rate may also have higher cellularity as a result. Finally, lower *TRATE* values in gliomas exposed to treatment can be explained with the notion that the enhancing regions in recurrent (R) gliomas are possibly characterized by a lower cellularity overall, due to a combination of malignant cells and treatment effects, including hyaline vasculopathy, reactive gliosis, and radiation necrosis, which were documented in histopathological reports in 50% of R-gliomas in our cohort. This explanation is also supported by higher values of other metrics reflecting the amplitude of EEC (i.e., ADC and *v*_*e*_) in our recurrent subcohort. Further studies longitudinally comparing *TRATE* and Δ*R*_1,ss_ values before and after chemoradiation are warranted to better understand the treatment-induced changes in these novel metrics, along with their potential role for treatment response assessment.

PSR values displayed similar group differences compared to TRATE, but with opposite direction, and these two metrics had a strong inverse correlation. Although PSR is easier to obtain, *TRATE* should be considered a *refined* measure of the balance between T_2_*- and T_1_-leakage effects compared to PSR, as it is measured in units and insensitive to acquisition parameters (i.e., FA and TE). Additionally, the pipeline for *TRATE* computation has the advantage of *separately* quantifying T_2_*- and T_1_-leakage effects (by computing Δ*R*_2,ss_* and Δ*R*_1,ss_, respectively), therefore providing additional information. Nevertheless, our results suggest that institutions where *TRATE* computation is not yet available may use PSR to obtain cytoarchitectural insights, with the *caveat* of its dependency upon TE and FA.

**Fig. 5:**
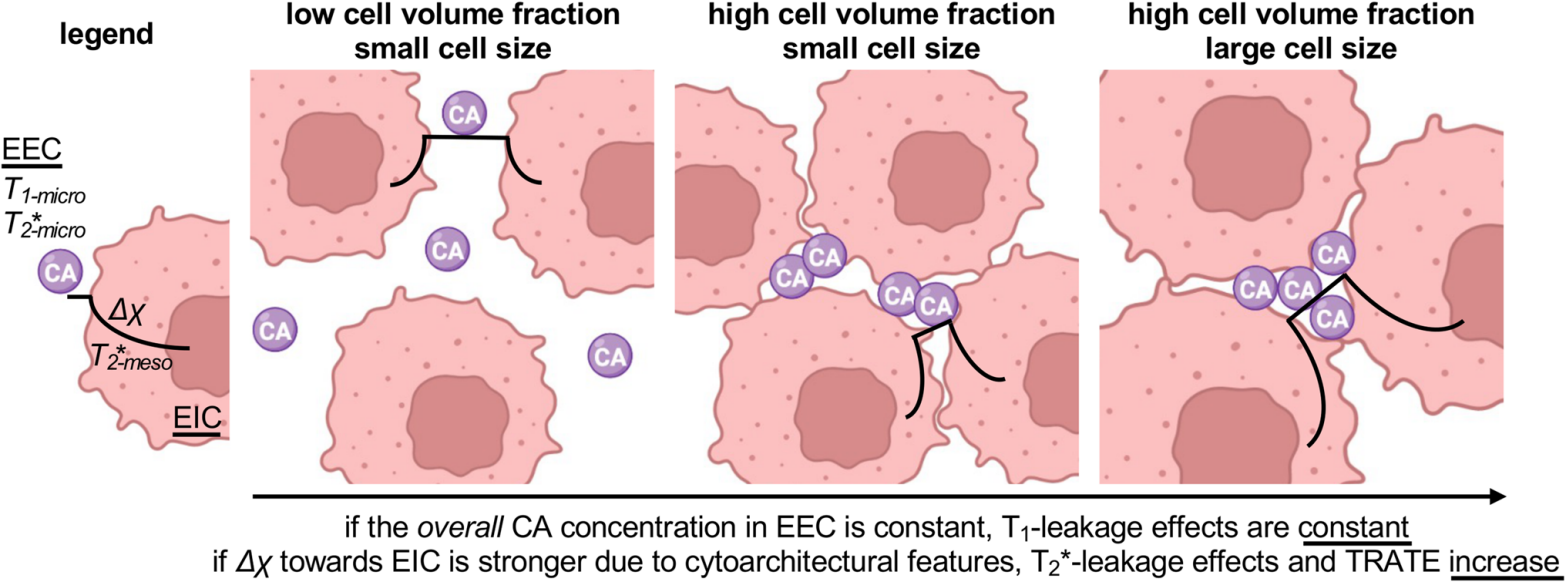
Schematic interpretation of contrast agent (CA) leakage effects in T_2_*-weighted DSC perfusion imaging. After leaking, CA induces T_1_ and T_2_* *microscopic* leakage effects in the extravascular extracellular compartment (EEC), as well as T_2_* *mesoscopic* leakage effects that take place also in the extravascular intracellular compartment (EIC) due to susceptibility gradients (*Δχ*) arising in the CA proximity. While microscopic effects only depend on CA concentration, mesoscopic effects are thought to be enhanced by a higher cell volume fraction and/or a larger cell size, possibly due to an increased CA packing in the proximity of cell membranes, which results in a stronger *Δχ* (the black lines represent the steepness of *Δχ*, which progressively increases from the left panel to the right panel, consistently with CA clustering). Conversely, ADC is sensitive to EEC amplitude but not to cytoarchitecture; therefore, ADC values in the right panel may be comparable to the central panel

A potential objection to the usefulness of *TRATE* is that ADC is a well-established proxy of cell density in gliomas [17, 57], and it is easier to obtain in the clinical setting. However, ADC values are thought to mainly reflect the amplitude of EEC, and also to be influenced by the extracellular matrix composition [58, 59]. Conversely, *TRATE* values are thought to depend on the steepness of the susceptibility gradients induced by CA molecules in EEC onto the extravascular intracellular compartment (EIC), which depends on the clustering of CA molecules in EEC and their proximity to cell membranes. Therefore, leakage effect measurements, as assessed by *TRATE* or PSR, provide a unique cytoarchitectural contrast that ultimately depends on the combination of cell volume fraction and cell size. This interpretation, depicted in Fig. 5 and elaborated in light of previous studies [32, 33], is also supported by our observation that the correlation between *TRATE* and ADC was only moderate in the TN-gliomas and non-significant in R-gliomas, and by Semmineh et al [33] reporting a low voxel-wise correlation between *TRATE* and ADC. Additionally, previous literature showed that PSR values performed better than ADC in some applications such as differential diagnosis [60], probably due to the unique contrast of leakage effect measurements, reflecting cytoarchitecture. To note, other studies have proposed to predict cell density with relaxometry [61] and deep learning methods [62], and to assess cell size with diffusion biophysical models [63]. As an overview, Table 5. reports a hypothesized pathophysiologic interpretation of dynamic SAGE-EPI metrics.

**Table 5:** Hypothesized pathophysiologic interpretation of dynamic SAGE-EPI metrics in gliomas

Biologic correlates	Underlying pathophysiologic event	MRI metrics
↑ Vascular density	Angiogenesis	↑ nrCBV
↑ Vascular permeability	BBB disruption linked to angiogenesis and/or treatment-induced effects	↑ *K*^trans^, ↑ Δ*R*_1,ss_
↑ Cell volume fraction *	Tumor proliferation	↑ TRATE, ↓ PSR, ↓ *v*_*e*_
↓ Cell size *	Tumor cell shrinkage following cytotoxic treatment	↓ TRATE, ↑ PSR

^*^TRATE and PSR differences due to cell volume fraction and cell size differences may also be exploited for differential diagnosis. Note that the proposed interpretations are partly based on speculation, and not all these correlates have been demonstrated. *BBB*, blood–brain barrier

This study has some limitations, including being a single-institution study. Future studies may compare *TRATE* values across institutions, to validate it as a parameter-insensitive leakage effect measurement compared to PSR, while being aware that *TRATE* values still depend on CA type and field strength. An immediate benchmark for this comparison would be the differential diagnosis, a well-established PSR application. Another limitation of this article is the lack of histopathological quantitative validation assessing *TRATE* association with cell volume fraction and cell size. Moreover, we did not perform a separate set of experiments to validate and compare DCE obtained from our pipeline with traditional DCE, because this would have required separate injections of contrast, with a study design similar to other articles evaluating DSC metrics with and without preload [31, 36]. The proposed DCE analysis differs from a traditional DCE because it is performed on T_1_w signal extrapolated from EPI acquisitions, and it has lower spatial resolution and a shorter acquisition time (~ 3 min vs ~ 5–6 min in typical DCE sequences optimized for brain tumors) [14, 64, 65]. EPI acquisitions result in more pronounced susceptibility artifacts in the proximity of tissue-air interfaces, constituting a limitation only for lesions located in temporal poles and fronto-basal gyri. A lower spatial resolution limits the assessment of subtle tumor heterogeneity, but does not impact our estimation of median *K*^trans^ and *v*_*e*_ within tumor tissue. The shorter acquisition time may affect the accuracy of DCE metrics and leakage effect metrics, since CA leakage is thought to reach an equilibrium in 5–10 min [33]. Future studies may explore dynamic SAGE-EPI with a longer acquisition time to solve this potential limitation. Future studies may also explore the potential validity of our methodology in non-enhancing gliomas, for which the utility of metrics related to CA extravasation (*K*^trans^, *v*_*e*_, Δ*R*_1,ss_, *TRATE*) is more ambiguous, since no gross CA extravasation is seen on T_1_w anatomical images. Additionally, it is worth mentioning that, while the proposed pipeline is feasible with a simpler dual-echo GE DSC, dynamic SAGE-EPI also contains additional echoes that can be used to perform additional vessel size imaging (VSI) [11] and vessel architecture imaging (VAI) [66]. Finally, this study was aimed at proposing a simultaneous analysis for multiple imaging metrics, rather than assessing nrCBV accuracy. Our proposed methodology *as it is* is not compliant with the current DSC guidelines, which advise for single-echo DSC using either 60° FA with preload or 30° FA without preload [67]. However, we employed a bidirectional leakage correction algorithm that minimizes the impact of pulse sequence parameters on nrCBV calculation [43]. If compliance with guidelines is desired, an easy solution would be to change dynamic SAGE-EPI FA to 30°, in order to obtain simultaneous guideline-compliant DSC, DCE, and leakage effect metrics with only one dose of contrast. This should not impact leakage effect measurements, since dual-echo computed signals (e.g., Δ*R*_2_*(*t*)) should be minimally impacted by pulse sequence parameters [31]. However, in our protocol, FA is set to 90° because lowering the FA would result in very low signal from the spin echo sequences included in dynamic SAGE-EPI, which would affect VSI and VAI. An alternative possible solution would be to compute nrCBV from dual-echo derived Δ*R*_2_*(*t*), an approach that has been shown to be as accurate as single-echo DSC with preload [31], and which may be eventually incorporated in future guidelines.

## Conclusions

We propose an image processing pipeline to generate perfusion, permeability, and novel leakage effect quantitative maps from a *single* dynamic SAGE-EPI sequence with a single bolus of contrast agent. This method can reduce scanning time and halve contrast agent administration compared to acquiring two separate sequences for perfusion and permeability imaging, and provides complementary leakage effect metrics. Among leakage effect metrics, Δ*R*_1,ss_ shows potential as a quantitative biomarker for blood–brain barrier breakdown, while *TRATE* represents a refined version of PSR, which may capture unique cytoarchitectural information dependent on cell volume fraction and cell size.

### Supplementary Information

Below is the link to the electronic supplementary material.

Supplementary file1 (PDF 531 KB)

## Abbreviations

ADC: Apparent diffusion coefficient
AIF: Arterial input function
BBB: Blood–brain barrier
CA: Contrast agent
DCE: Dynamic contrast enhanced
DSC: Dynamic susceptibility contrast
DTI: Diffusion tensor imaging
DWI: Diffusion-weighted imaging
E1: Echo 1 of dynamic SAGE-EPI
E2: Echo 2 of dynamic SAGE-EPI
EEC: Extravascular extracellular space
EIC: Extravascular intracellular space
FA: Flip angle
FLAIR: Fluid-attenuated inversion recovery
GE: Gradient echo
IDH: Isocitrate dehydrogenase
IDH^m^: IDH-mutant
IDH^wt^: IDH wild-type
IV: Intravascular space
K^trans^: Volume transfer constant
MRI: Magnetic resonance imaging
nrCBV: Normalized relative cerebral blood volume
PSR: Percentage of signal recovery
R: Recurrent
ROI: Region of interest
SAGE-EPI: Spin-and-gradient-echo echoplanar imaging
TE: Echo time
TN: Treatment-naïve
TR: Repetition time
TRATE: Transverse relaxation at tracer equilibrium
v_e_: Extravascular compartment
ΔR_1__,ss_: Change in longitudinal relaxation rate over time compared to baseline
ΔR_2__,ss_*: Change in transverse relaxation rate over time compared to baseline

## Declarations

### Guarantor

The scientific guarantor of this publication is Benjamin M Ellingson, University of California Los Angeles.

### Statistics and biometry

No complex statistical methods were necessary for this paper.

### Study subjects or cohorts overlap

Some study subjects or cohorts have been previously reported in:

Chakhoyan et al 2018, Validation of vessel size imaging (VSI) in high-grade human gliomas using magnetic resonance imaging, image-guided biopsies, and quantitative immunohistochemistry.

### Methodology

• prospective

• diagnostic or prognostic study

• performed at one institution
