# Supplementary material for: Simultaneous quantification of perfusion, permeability, and leakage effects in brain gliomas using dynamic spin-and-gradient-echo echoplanar imaging MRI
Source: Eur Radiol. 2023 Oct 26;34(5):3087–101. doi: 10.1007/s00330-023-10215-z (PMC11045669; doi:10.1007/s00330-023-10215-z)
Supplement: Supplementary file 1 — Supplementary file1 (PDF 531 KB) [file 330_2023_10215_MOESM1_ESM.pdf]

Simultaneous quantification of perfusion, permeability, and leakage effects in brain gliomas using dynamic spin-and-gradient-echo echoplanar imaging MRI

Electronic supplementary material

Suppl.Eq.1: (Stokes et al., 2021)

$$\Delta R_2^*(t) = \frac{1}{TE_2 - TE_1}\left[\ln\left(\frac{S_{TE1}(t)}{S_{TE1}(0)}\right) - \ln\left(\frac{S_{TE2}(t)}{S_{TE2}(0)}\right)\right]$$

where:

- $\Delta R_2^*(t)$ [s^{-1}] is the change in the transverse relaxation rate over time compared to baseline;
- $S_{TE1}(t)$ and $S_{TE2}(t)$ are the signals of E1 and E2 (respectively) over time;
- $S_{TE1}(0)$ and $S_{TE2}(0)$ are the prebolus signals of E1 and E2 (respectively) computed by averaging the initial 10 timepoints of the corresponding echo;
- TE_1 and TE_2 are the TEs of E1 and E2 (respectively) in seconds (in our case, 0.014 s and 0.0341 s, respectively).

Suppl.Eq.2: (Stokes et al., 2021)

$$T_1w(t) = S_{TE1}(t)\left(\frac{S_{TE1}(t)}{S_{TE2}(t)}\right)^{\frac{TE1}{(TE2-TE1)}}$$

where:

- $T_1w(t)$ is the purely T_1-weighted contribution to the DSC acquisition, obtained by extrapolating the estimated signal at TE=0 s;
- all other terms are as in Suppl.Eq.1.

Suppl.Eq.3:

$$\Delta T_2^*(t) = T_2^*(t) - T_2^*(0)$$

with:

$$T_2^*(t) = \frac{1}{-\frac{1}{TE_2 - TE_1}\left[\ln(S_{TE2}(t)) - \ln\left(S_{TE1}(t)\right)\right]}$$

and:

$$T_2^*(0) = \frac{1}{-\frac{1}{TE_2 - TE_1}\left[\ln(S_{TE2}(0)) - \ln\left(S_{TE1}(0)\right)\right]}$$

where:

- $T_2{}^*(t)$ [s] is the transverse relaxation over time;
- $\Delta T_2{}^*(t)$ [s] is the change in $T_2{}^*(t)$ compared to baseline;
- $T_2{}^*(0)$ is the prebolus $T_2{}^*$;
- all other terms are as in Suppl.Eq.1-2.

Suppl.Eq.4:
(https://www.rsna.org/research/quantitative-imaging-biomarkers-alliance; https://github.com/OSIPI/DCE-DSC-MRI_CodeCollection)

$$\Delta R_1(t) = R_1(t) - \frac{1}{T_{10}}$$

with:

$$R_1(t) = \frac{-1}{TR}\ln\left(\frac{1 - A}{1 - \cos(\propto) * A}\right)$$

with:

$$A = \left(\frac{1 - E_{10}}{1 - \cos(\propto) * E_{10}}\right)\left(\frac{T_1w(t)}{T_1w(0)}\right)$$

with:

$$E_{10} = e^{\frac{-TR}{T_{10}}}$$

where:

- $R_1(t)$ [s^{-1}] is the longitudinal relaxation over time;
- $\Delta R_1(t)$ [s^{-1}] is the change in $R_1(t)$ compared to baseline;
- T_{10} is the fixed estimated pre-bolus quantitative T_1 for brain tissue at 3T based on literature reports – we picked 1.4 s as in (Conte et al., 2019);
- TR is the TR of the DCE datasets, obtained as $TR_{DCE} = TR_{DSC} / [(y_{DCE})(z_{DCE})]$, since the DCE dataset is derived from a dynamic SAGE-EPI;
- $T_1w(0)$ is the prebolus T_1w contribution, computed by averaging the initial 10 timepoints of $T_1w(t)$;
- α is the flip angle (in our case, 90°);
- all other terms are as in Suppl.Eq.1-3.

Suppl.Eq.5:

$$TRATE = r^*_{2,ss} = \frac{\Delta R^*_{2,ss}}{C_{ss}}$$

where:

- $r_{2,ss}$* [$mM^{-1}s^{-1}$] is the transverse relaxivity at tracer equilibrium (*TRATE*);
- $\Delta R_{2,ss}$* [s^{-1}] is post-bolus ΔR_2* at the steady state, computed by averaging the last 10 volumes of $\Delta R2^*_{(t)}$ [s^{-1}];
- C_{ss} [mM] is the estimated contrast agent concentration at the steady state, computed by averaging the last 10 volumes of by C(*t*);

with:

$$C(t) = \frac{\Delta R_1(t)}{r_1}$$

where:

- C(*t*) [mM] is the estimated contrast agent concentration over time;
- r_1 is the relaxivity of Gadobutrol at 3T, set to 5.0 $mM^{-1}s^{-1}$ as computed by (Rohrer et al., 2005) and reported by the American College of Radiology (https://www.acr.org)

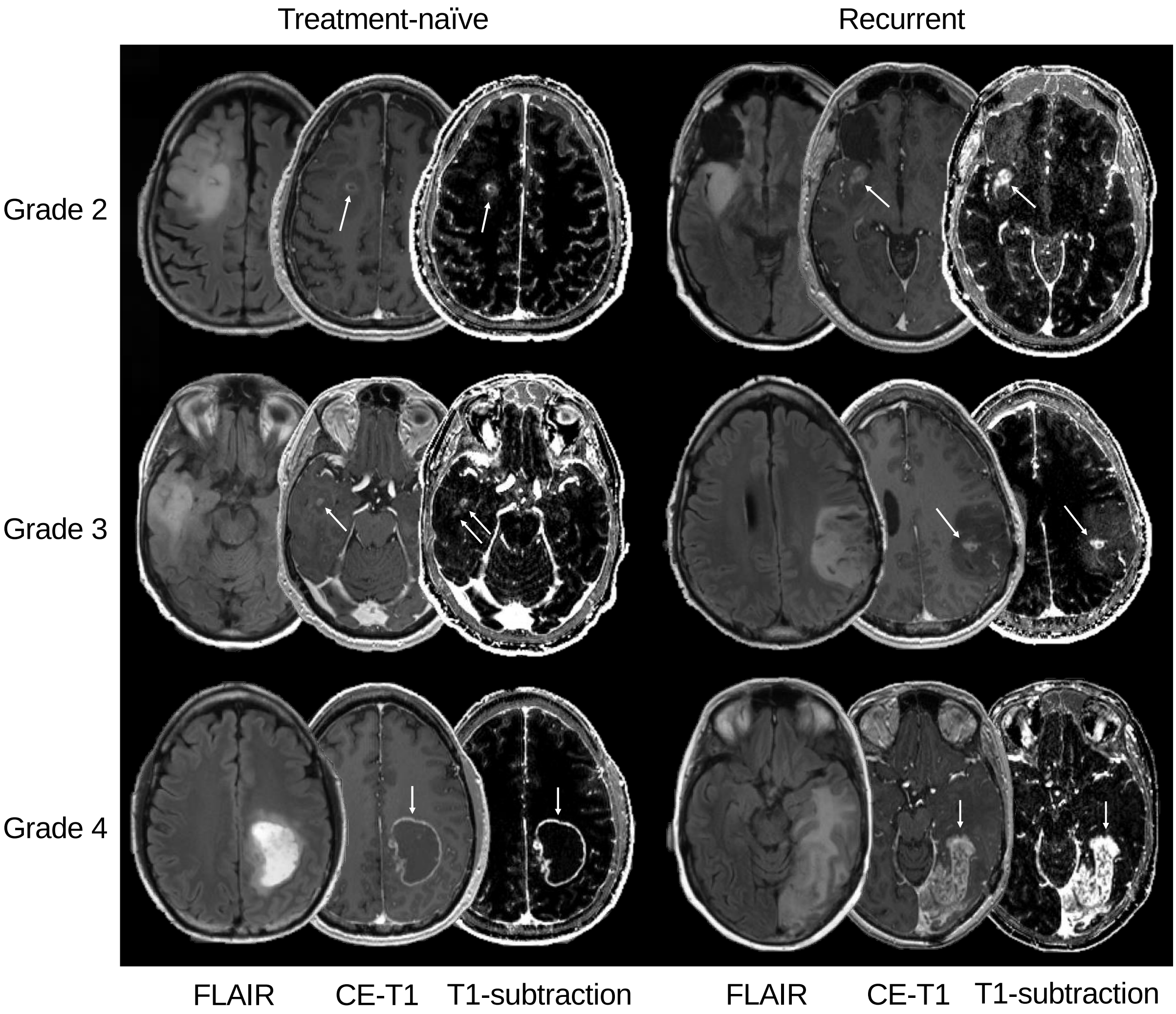

Suppl.Fig.1. Conventional MRI appearance of six representative cases randomly selected from the study cohort. The arrows point at the areas of enhancement. CE-T1 = contrast-enhanced T1; T1-subtraction = T1 subtraction maps obtained by removing normalized pre-contrast T1 signal from normalized post-contrast T1 signal.

References

Conte, G.M., Altabella, L., Castellano, A., Cuccarini, V., Bizzi, A., Grimaldi, M., Costa, A., Caulo, M., Falini, A., Anzalone, N., 2019. Comparison of T1 mapping and fixed T1 method for dynamic contrast-enhanced MRI perfusion in brain gliomas. Eur. Radiol. 29, 3467–3479. https://doi.org/10.1007/s00330-019-06122-x

Rohrer, M., Bauer, H., Mintorovitch, J., Requardt, M., Weinmann, H.-J., 2005. Comparison of magnetic properties of MRI contrast media solutions at different magnetic field strengths. Invest. Radiol. 40, 715–724. https://doi.org/10.1097/01.rli.0000184756.66360.d3

Stokes, A.M., Bergamino, M., Alhilali, L., Hu, L.S., Karis, J.P., Baxter, L.C., Bell, L.C., Quarles, C.C., 2021. Evaluation of single bolus, dual-echo dynamic susceptibility contrast MRI protocols in brain tumor patients. J. Cereb. blood flow Metab. Off. J. Int. Soc. Cereb. Blood Flow Metab. 41, 3378–3390. https://doi.org/10.1177/0271678X211039597
